# Menthol Cigarette Smoke Induces More Severe Lung Inflammation Than Non-menthol Cigarette Smoke Does in Mice With Subchronic Exposure – Role of TRPM8

**DOI:** 10.3389/fphys.2018.01817

**Published:** 2018-12-18

**Authors:** An-Hsuan Lin, Meng-Han Liu, Hsin-Kuo Ko, Diahn-Warng Perng, Tzong-Shyuan Lee, Yu Ru Kou

**Affiliations:** ^1^Department of Physiology, School of Medicine, National Yang-Ming University, Taipei, Taiwan; ^2^Department of Chest Medicine, Taipei Veterans General Hospital, Taipei, Taiwan; ^3^Graduate Institute and Department of Physiology, College of Medicine, National Taiwan University, Taipei, Taiwan

**Keywords:** cigarette smoke, menthol, TRPM8, reactive oxygen species, MAPKs, lung inflammation, lung epithelial cells

## Abstract

In smokers with chronic obstructive pulmonary disease, more severe lung inflammation is associated with menthol cigarette smoking compared to non-menthol cigarette smoking. However, the mechanisms remain unclear. Menthol is an activator of transient receptor potential melastatin-8 (TRPM8), which is also sensitive to reactive oxygen species (ROS). Our recent *in vitro* study demonstrated that the extracts of menthol cigarette smoke (M-CS) can induce greater ROS-sensitive, TRPM8-mediated, mitogen-activated protein kinase (MAPK)-dependent inflammatory responses in lung epithelial cells than the extracts of non-menthol cigarette smoke (Non-M-CS) can. In this study, we tested the hypothesis that M-CS can induce more severe lung inflammation than Non-M-CS can via the additional action of menthol in M-CS on epithelial and lung TRPM8 in mice. Compared with Non-M-CS exposure, subchronic M-CS exposure for 7 days up-regulated the epithelial and lung expression of TRPM8, induced more vigorous activation of epithelial and lung MAPKs, and caused more severe lung inflammation. The MAPK activation was evidenced by the increased expression of phosphor-extracellular signal-regulated and phosphor-c-Jun N-terminal kinases. The lung inflammation was evidenced by pathohistological findings and increases in several inflammatory indices. Moreover, treatment with a TRPM8 antagonist (*N*-(3-aminopropyl)-2-{[(3-methylphenyl)methyl]oxy}-*N*-(2-thienylmethyl)benzamide; AMTB) greatly suppressed the MAPK activation and lung inflammation induced by Non-M-CS and M-CS, and the residual responses to these two types of CS did not differ. Conversely, the levels of biomarkers of acute CS exposure (20 min), including carboxyhemoglobin and cotinine (a nicotine metabolite) in blood plasma, and superoxide and hydrogen peroxide (two major types of ROS) in bronchoalveolar lavage fluid, did not show significant differences in the mice with Non-M-CS and M-CS exposure. We concluded that M-CS could induce greater TRPM8-mediated activation of MAPKs and lung inflammation than Non-M-CS could in mice with subchronic exposure. The augmented inflammatory effects of M-CS are unlikely due to a larger total amount of CS inhaled, but may be caused by an additional stimulation of epithelial and lung TRPM8 by menthol in M-CS. A common stimulant (presumably ROS) generated by both CS types may also stimulate TRPM8, activate MAPKs, and induce lung inflammation because treatment with AMTB could reduce these responses to Non-M-CS.

## Introduction

The use of menthol cigarettes in the United States has increased in recent years despite the concern regarding the more detrimental effects of menthol cigarette smoking relative to those of non-menthol cigarette smoking (Hoffman, 2011; Besaratinia and Tommasi, 2015). Thus, much interest has been focused on the investigation into the health effects of menthol compared with non-menthol cigarettes (Werley et al., 2007; Hoffman, 2011). Recently, a large-scale cohort study reported that more frequent severe exacerbations of chronic obstructive pulmonary disease were observed in menthol cigarette smokers compared to non-menthol cigarette smokers (Park et al., 2015), suggesting that this patient population may result in more severe lung inflammation when they use menthol cigarettes. A related hypothesis is that menthol cigarette smokers may inhale a larger total volume of cigarette smoke (CS) into their lungs due to the counter-airway irritation effect of menthol, which leads to more adverse effects compared with those of non-menthol cigarettes (Hoffman, 2011; Lawrence et al., 2011; Besaratinia and Tommasi, 2015). However, this hypothesis is not supported by results from studies using animals (Gaworski et al., 1997; Ha et al., 2015) and smokers (Caraballo et al., 2001; Heck, 2009; Hoffman, 2011; Lawrence et al., 2011; Strasser et al., 2013); these studies measured the smoking topography and blood biomarkers of smoke exposure. Thus, the pathophysiological mechanisms underlying the more severe lung inflammation associated with menthol cigarette smoking remain obscure.

Menthol is an activator of transient receptor potential melastatin-8 (TRPM8; also known as the cold receptor) that is primary expressed in the primary afferent neurons (Journigan and Zaveri, 2013). Upon activation of neuronal TRPM8, menthol can produce the sensation of airway cooling (Fisher, 2011). However, many studies have reported that TRPM8 is also expressed in non-neuronal lung cells (Yang et al., 2006; Johnson et al., 2009; Journigan and Zaveri, 2013; Zhang et al., 2016), including lung epithelial cells (Sabnis et al., 2008a,b; Li et al., 2011; Lin et al., 2017), the first target in the airways for the direct insult of CS. To this end, TRPM8 activation by cold temperature or menthol has been shown to induce inflammatory responses from lung epithelial cells (Sabnis et al., 2008a,b; Li et al., 2011; Lin et al., 2017). More important, our recent *in vitro* study (Lin et al., 2017) demonstrated that the extracts of menthol cigarette smoke (M-CS) can induce greater reactive oxygen species (ROS)-sensitive, TRPM8-mediated, mitogen-activated protein kinase (MAPK)-dependent inflammatory responses in human lung epithelial cells than the extracts of non-menthol cigarette smoke (Non-M-CS) can. We also showed that a dual action of both ROS and menthol in M-CS on lung epithelial TRPM8 may be responsible for these augmented effects (Lin et al., 2017). Collectively, these observations suggest the possibility that in addition to CS, the menthol in the smoke may act as another stimulus via its action on TRPM8 to induce lung inflammation. However, *in vivo* findings to support this hypothesis are still lacking.

In the present study, we hypothesized that M-CS could induce more severe lung inflammation than Non-M-CS could via the additional action of menthol on TRPM8. In testing this hypothesis, we employed a murine model of CS exposure (Tang et al., 2011; Liu et al., 2014; Wu et al., 2014) to compare various indices of lung inflammation and activation of MAPKs in lung tissues induced by subchronic exposure to Non-M-CS and M-CS. We used a selective TRPM8 antagonist (*N*-(3-aminopropyl)-2-{[(3-methylphenyl) methyl]oxy}-*N*-(2-thienylmethyl) benzamide; AMTB) (Journigan and Zaveri, 2013) as the intervention to assess the role of TRPM8 in these responses. We also measured several biomarkers of CS exposure to reflect the total amounts of CS inhaled by mice during acute exposure to Non-M-CS or M-CS.

## Materials and Methods

### Reagents

Macrophage inflammatory protein 2 (MIP-2) and AMTB hydrochloride were measured with an ELISA kit purchased from R&D Systems (Minneapolis, MN, United States). Cotinine was also measured with an ELISA kit purchased from Calbiotech (La Jolla, CA, United States). From Abcam (Cambridge, MA, United States), we purchased the rabbit Ab against TRPM8 and 4-hydroxynonenal (4-HNE). From Cell Signaling (Beverly, MA, United States), we purchased the rabbit Ab against c-Jun N-terminal kinases (JNK). From BD Biosciences (San Jose, CA, United States), we purchased the mouse Ab against phosphor-JNK (p-JNK) was purchased. From Santa Cruz Biotechnology (Santa Cruz, CA, United States), we purchased the mouse Abs against extracellular signal-regulated kinase (ERK) and phosphor-ERK (p-ERK). From Sigma-Aldrich (St. Louis, MO, United States), we purchased the mouse Ab against α-tubulin. From Molecular Probes (Eugene, OR, United States), we purchased the membrane-permeable probe hydroethidine (HE) and dichlorofluorescein diacetate (DCFH-DA) were purchased.

### Subchronic CS Exposure in Mouse Model

The Animal Care and Use Committee of the National Yang-Ming University approved all animal experiments performed in this study. For the subchronic exposure study, male C57BL/6J mice aged 8 weeks (National Laboratory Animal Center, Taipei, Taiwan) were randomly assigned to three groups subjected to exposure to air, Non-M-CS, or M-CS in some experiments. In other experiments, mice were randomly assigned to five groups subjected to exposure to air, Non-M-CS, or M-CS. Among these groups, two groups of mice were treated with AMTB (a TRPM8 antagonist; 25 mg/kg, i.p.), and three other groups of mice were treated with PBS (vehicle control, i.p.). Treatments with PBS and AMTB were given every 2 days during the 7-day CS exposure. The dose and route of administration of the AMTB treatment were adopted from a previous study (Mueller-Tribbensee et al., 2015). Thus, mice were divided into five experimental groups: Air, Non-M-CS, Non-M-CS + AMTB, M-CS, and M-CS + AMTB. The detail methods of CS exposure in this mouse model of CS exposure have been described elsewhere (Tang et al., 2011). We used West Rich Blue non-menthol and Marlboro Black Menthol cigarettes in this study. These two brands were employed because each cigarette has the same diameters (0.8 cm), lengths without a filter (5.6 cm), nicotine levels (0.6 mg), and tar levels (8 mg). The amounts of tobacco (approximately 600 mg) in each cigarette of these two brands were also similar. The mice were placed in an exposure chamber during each CS exposure. A total of 750 mL of fresh CS generated from 1.5 cigarettes was delivered to the chamber. The CS passed out of the chamber via four exhaust holes (1 cm) on the side panels. For each CS exposure, the particle concentration inside the exposure chamber was approximately 625 mg/m^3^ initially but decreased overtime due to the CS passing out of the chamber via the exhaust holes. During the exposure, the mice were conscious and breathed spontaneously in the chamber for 10 min. After exposure, the mice were transferred to a new cage and allowed to inspire air normally. The mice were exposed at 10:00 and 16:00 each day for 7 days. The control animals underwent identical procedures in another chamber but were only exposed to air. Animals were given *ad libitum* access to food and water, and the averaged body weights did not vary among the study groups after the 7-day exposure.

### Sampling for Bronchoalveolar Lavage Fluid (BALF) and Lung Tissues

The mice were sacrificed with CO_2_ at the end of each experiment in the subchronic CS exposure study. The mice received a middle thoracotomy with ligation of the left lung. The right lung was lavaged four times with warm PBS (0.6 mL) containing a complete protease inhibitor cocktail (Roche Diagnostics, Mannheim, Germany). Then, the BALF samples were centrifuged at a condition of 350 × *g* for 5 min at 4°C. The supernatant of the first lavage fluid was then stored at -80°C for later analysis of total protein using an assay (Bio-Rad Laboratories, Inc., Hercules, CA, United States). Cell counting was performed after the process when the cell pellets of the BALF samples were resuspended in PBS. The left lung was fixed with 4% paraformaldehyde and embedded in paraffin. The tissues of the right lung were then stored at -80°C for subsequent analysis.

### Histological Assessments

The tissue blocks were cut into 8 μm sections, which were subsequently deparaffinized, rehydrated, hematoxylin and eosin (H&E) stained. These sections were viewed by a pathologist who was blinded to the treatment to perform the histological assessments. Each histological characteristic was scored on a scale of 0 (normal) to 5 (maximal). The severity of inflammation was graded according to the following scale: no inflammation = 0; inflammatory characteristic shown in 20% of the field = 1; inflammatory characteristic shown in 40% of the field = 2; inflammatory characteristic shown in 60% of the field = 3; inflammatory characteristic shown in 80% of the field = 4; and diffused inflammation = 5. The lung inflammatory score was categorized according to the sum of the scores for the infiltration cell numbers and damage level, including the thickening of alveolar walls and epithelium. This grading method has been used in our previous studies (Liu et al., 2014; Lin et al., 2015).

### Immunohistochemical Assessments

Tissue blocks were cut into 8 μm sections. The sections were deparaffinized, rehydrated, and covered with 3% H_2_O_2_ for 10 min. After being blocked with bovine serum albumin, the slides were incubated with primary antibodies for 1 h at 37°C and with corresponding secondary antibodies for an additional hour. Antigenic sites were visualized by the addition of DAB. The slides were counterstained with hematoxylin. The quantification of the expression levels of p-ERK and p-JNK in epithelial cells positive for immunostaining was performed using an image analysis system (Image-Pro Plus 4.5; Media Cybernetics, Bethesda, MD, United States), as described previously (Yang et al., 2008).

### *En Face* Immunostaining of the Entire Lung

The method for the *en face* immunostaining of the entire lung has been described previously (Lin et al., 2015). C57BL/6J mice aged 8 weeks were sacrificed with CO_2_, and a middle thoracotomy was performed. The lungs were then infused with 4% paraformaldehyde (0.8 mL) overnight. After infusion with 3% H_2_O_2_ and blockage with bovine serum albumin, the lungs were infused with IgG or TRPM8 Ab overnight at 4°C and then with corresponding HRP-conjugated secondary Ab for 1 h. The antigenic sites were visualized by the addition of DAB. The lung was then soaked in xylene, and its image was photographed by a digital camera (Sanyo, Osaka, Japan).

### Western Blot

Lungs were isolated and lysed by lysis buffer containing 1% Triton X-100, Tris (50 mM) pH 7.5, EDTA (5 mM), NaCl (300 mM), PMSF (1 mM), leupeptin (10 μg/mL) and aprotinin (10 μg/mL). Aliquots of lysate protein were separated on 8–12% SDS-PAGE and then transferred to membranes. Specific protein on the membrane was immunoblotted with primary antibody, then horseradish peroxidase-conjugated secondary antibody. The specific protein bands were detected by using enzyme-linked chemiluminescence detection kit (PerkinElmer, Waltham, MA, United States) and quantified by Imagequant 5.2 (Healthcare Bio-Sciences, Philadelphia, PA, United States).

### Measurement of MIP-2 Concentration

According to the manufacturers’ instructions, the ELISA methods were used to measure the concentrations of MIP-2 in BALF in the lung tissue samples.

### Measurement of 4-HNE Level

The Western blot analysis was used to measured the level of 4-HNE modified proteins in the lung tissue samples. The levels of the product of lipid peroxidation were used to reflect the levels of oxidative stress (Rahman et al., 2002).

### Measurement of Biomarkers for Acute CS Exposure

For the study of acute exposure to CS, three additional groups of mice were exposed to air, Non-M-CS, or M-CS for 20 min. At the end of each experiment, the mice were euthanized with CO_2_, and arterial blood and BALF samples were obtained. The carboxyhemoglobin (HbCO) levels were measured by a blood gas system (Siemens Healthcare Diagnostics, Erlangen, German). The cotinine concentrations (a nicotine metabolite) in blood plasma were assessed with an ELISA kit according to the manufacturer’s instructions. The membrane-permeable probes HE and DCFH-DA were used to assess the levels of superoxide and hydrogen peroxide, respectively (Benov et al., 1998; Myhre et al., 2003), in the BALF by using methods that have been described previously (Liu et al., 2005). Briefly, the supernatant of the first BALF sampled from all study groups was incubated with 10 μm HE and 20 μm DCFH-DA at 37°C for 15 min. The ROS amount generated in the BALF could be reflected by the fluorescent intensities. The fluorescence intensities of the BALF samples were then analyzed using a multilabel counter (PerkinElmer, Waltham, MA, United States).

### Statistical Analysis

The results are presented as mean ± SEM. The normal distribution of data was checked by the Kolmogorov–Smirnov test. The statistical evaluations involved one-way ANOVA, followed by Tukey’s test for multiple comparisons when appropriate. Differences were considered statistically significant at *p* < 0.05.

## Results

### M-CS Induces Higher Lung Expression of TRPM8 Than Non-M-CS Does in Mice With Subchronic Exposure

**FIGURE 1:**
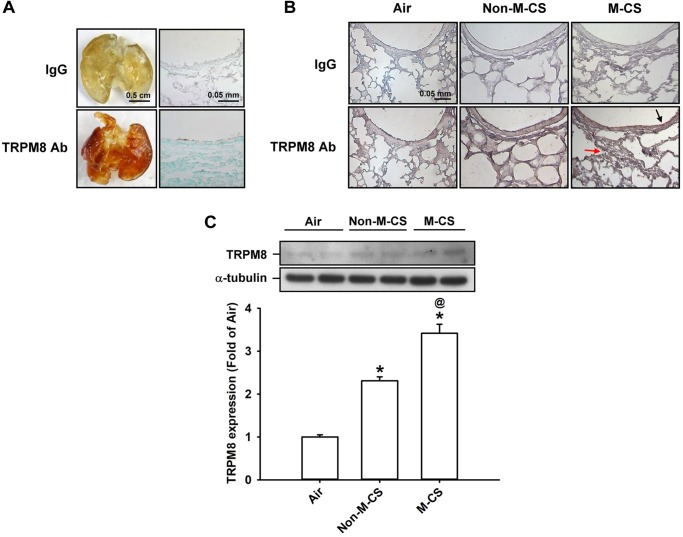
Menthol cigarette smoke (M-CS) induces higher lung expression of TRPM8 than non-menthol cigarette smoke (Non-M-CS) does in mice with subchronic exposure. Mice were exposed to air, M-CS, or Non-M-CS for 7 days. **(A)** Representative images showing the *en face* immunostaining with a TRPM8 antibody using whole lungs (left panels) and lung sections (right panels) obtained from air-exposed mice. The specificity of the immunostaining was confirmed using an IgG-negative control. **(B)** Representative images showing immunostaining with a TRPM8 antibody of the lung sections obtained from air- and CS-exposed mice. The magnification of each panel was 400×. The black arrow indicates the epithelium and the red arrow indicates inflammatory cell infiltration. **(C)** Western blot analysis showing expression levels of TRPM8 in the lung tissues obtained from air- and CS-exposed mice. Data in each group are mean ± SEM from nine mice. ^∗^*p* < 0.05 vs. the air-exposed group; ^@^*p* < 0.05 vs. the Non-M-CS exposed group.

We first studied the epithelial and lung expression of TRPM8 in mice. In the air-exposed animals, *en face* immunostaining showed positive staining for TRPM8 across the whole lungs of the mice. The geometry of the airways could be clearly visualized in the mice (Figure 1A). An analysis of the mouse lung sections obtained after *en face* immunostaining indicated that the lung epithelial cells also displayed positive staining for TRPM8 (Figure 1A). Further immunohistochemical analysis showed that a stronger positive staining for TRPM8 was observed in the epithelial cells of the lung sections of the mice that had been subchronically exposed to Non-M-CS or M-CS for 7 days than in those of the air-control mice (Figure 1B). In addition, the positive staining for TRPM8 was stronger in the epithelial cells of the lung sections of the M-CS-exposed mice than in those of the Non-M-CS-exposed mice (Figure 1B). Quantitative analysis revealed that the increase in the relative expression level of TRPM8 measured as positive cells by immunostaining for the M-CS-, Non-M-CS-, and air-exposed groups were 5 ± 0.1 (*n* = 9), 2.2 ± 0.1 (*n* = 9), and 1.0 ± 0.1 (*n* = 9) folds, respectively; each was significantly different from the others. Furthermore, Western blot analysis (Figure 1C) revealed that the expression of TRPM8 was significantly higher in the lung tissues of the M-CS-exposed mice than in those of the Non-M-CS-exposed mice. At the same time, the expression of TRPM8 was significantly higher in the lung tissues of the Non-M-CS-exposed mice than in those of the air-exposed mice.

### M-CS Induces More Severe Lung Inflammation Than Non-M-CS Does in Mice With Subchronic Exposure — Role of TRPM8

**FIGURE 2:**
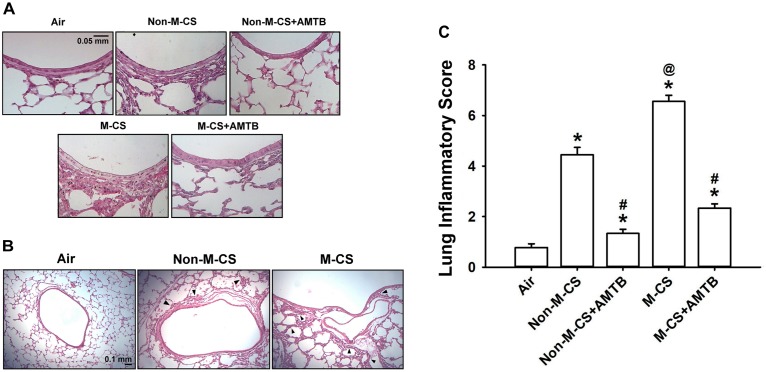
Menthol cigarette smoke (M-CS) induces more severe lung inflammation than non-menthol cigarette smoke (Non-M-CS) does in mice with subchronic exposure. Five groups of mice were exposed to air, Non-M-CS, or M-CS for 7 days. Mice also received daily treatment with AMTB (a TRPM8 antagonist; 25 mg/kg body weight) or saline (the vehicle) by intraperitoneal injection during the 7-day exposure. **(A)** Representative images of H&E-stained lung sections obtained from five study groups. The magnification of each panel was 400×. **(B)** Representative images of H&E-stained lung sections obtained from three study groups. The magnification of each panel was 100×. Arrows indicate inflammatory cell infiltration. **(C)** Lung inflammatory scores were calculated according to the sum of the levels of cell infiltration and damage stages assessed from the lung sections. Data in each group are mean ± SEM from nine mice. ^∗^*p* < 0.05 vs. the air-exposed group; ^@^*p* < 0.05 vs. the Non-M-CS-exposed group; ^#^*p* < 0.05 vs. the same type of CS group with vehicle treatment. Note that the lung inflammation induced by both types of CS was suppressed by treatment with AMTB.

**FIGURE 3:**
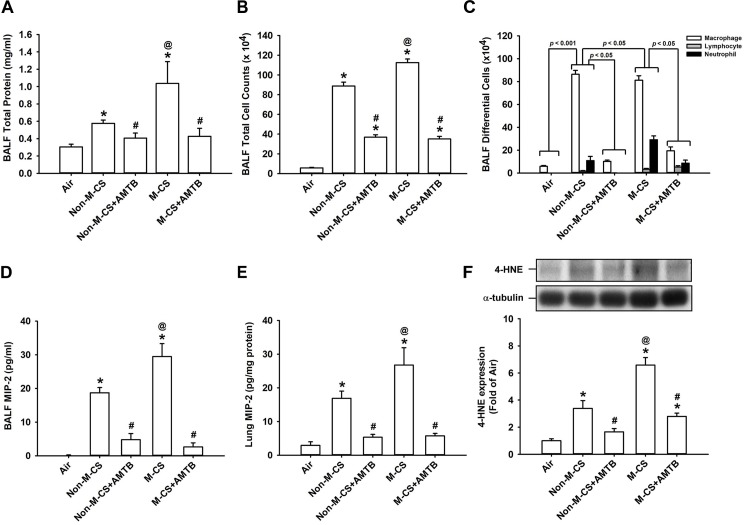
Treatment with a TRPM8 antagonist (AMTB) suppresses the lung inflammation induced by M-CS or Non-M-CS in mice with subchronic exposure. See Figure 2 caption for further explanations of the treatments in the five study groups of mice. Total protein content **(A)**, total cell count **(B)**, differential cell count **(C)**, and levels of MIP-2 **(D)** in bronchoalveolar lavage fluid (BALF) were measured to serve as inflammatory indices of lung inflammation. Levels of MIP-2 **(E)** and 4-HNE **(F)** in the lung tissues were also analyzed with ELISA and Western blot, respectively, to provide additional indices. 4-HNE represents 4-hydroxynonenal (a major end product of lipid peroxidation reflecting the level of oxidative stress). Data in each group are mean ± SEM from nine **(A–E)** or six mice **(F)**. ^∗^*p* < 0.05 vs. the air-exposed group; ^@^*p* < 0.05 vs. the Non-M-CS-exposed group; ^#^*p* < 0.05 vs. the same type of CS group with vehicle treatment. Note that the increased inflammatory indices induced by M-CS were higher than those induced by Non-M-CS, but these increased inflammatory indices induced by both types of CS were significantly reduced to similar levels by treatment with AMTB.

**FIGURE 4:**
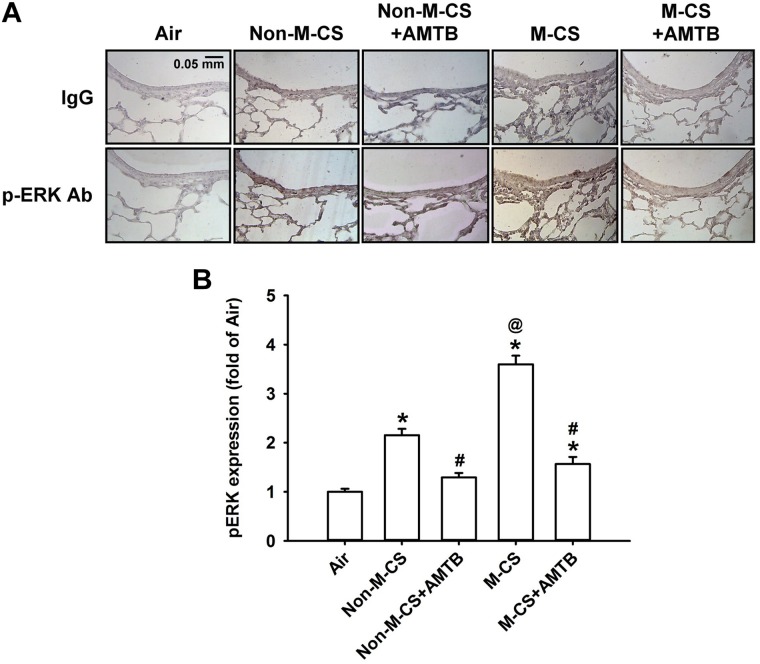
Menthol cigarette smoke induces higher lung epithelial expression of p-ERK than Non-M-CS does in mice with subchronic exposure. p-ERK represents phosphor-extracellular signal-regulated kinase. See Figure 2 caption for further explanations of the treatments in the five study groups of mice. **(A)** Representative images showing immunostaining with a p-ERK antibody of the lung sections obtained from the five groups of mice. Specificity of immunostaining was confirmed using an IgG-negative control. The magnification of each panel was 400×. **(B)** Quantification of the expression levels of epithelial p-ERK in the lung sections with immunostaining. Data in each group are mean ± SEM from six mice. ^∗^*p* < 0.05 vs. the air-exposed group; ^@^*p* < 0.05 vs. the Non-M-CS-exposed group; ^#^*p* < 0.05 vs. the same type of CS group with vehicle treatment. Note that the increased expression of epithelial p-ERK induced by M-CS was higher than that induced by Non-M-CS, but the increased expressions induced by both types of CS were significantly reduced to similar levels by treatment with AMTB.

To assess the role of TRPM8, we studied the CS-induced lung inflammation and suppressive effects of AMTB (a TRPM8 antagonist) on inflammatory responses. A histological evaluation of the lung sections of mice with M-CS exposure revealed presence of abnormal re-epithelialization, thickening of alveolar walls, and infiltration of inflammatory cells (Figures 2A,B). All of these changes were found to be less in the mice with Non-M-CS exposure than in those with M-CS exposure (Figures 2A,B). These images of lung sections with different magnifications (Figures 2A,B) were then used to assess lung inflammatory scores. The differences in the degree of lung inflammation of the mice subjected to these two types of CS exposure were confirmed by comparing the group data in terms of lung inflammatory scores (Figure 2C). Further analysis indicated that relative to the air-exposed mice, those with either type of CS exposure were found to show increases in BALF for total protein levels (Figure 3A; an index of increased lung vascular permeability), cell counts (Figure 3B), differential cell counts (Figure 3C), and MIP-2 levels (Figure 3D; a potent inflammatory chemokine), as well as increases in lung tissue samples for the levels of MIP-2 (Figure 3E) and 4-HNE (Figure 3F; a biomarker of oxidative stress). All these inflammatory indices were significantly higher in the mice with M-CS exposure than in those with Non-M-CS exposure (Figure 3). More important, these pathohistological manifestations (Figure 2A) and increases in lung inflammatory scores (Figure 2B) and in various lung inflammatory indices (Figure 3) induced by both types of CS were significantly reduced to a similar level by treatment with AMTB.

### M-CS Induces Higher Lung Expression of p-ERK or p-JNK Than Non-M-CS Does in Mice With Subchronic Exposure – Role of TRPM8

**FIGURE 5:**
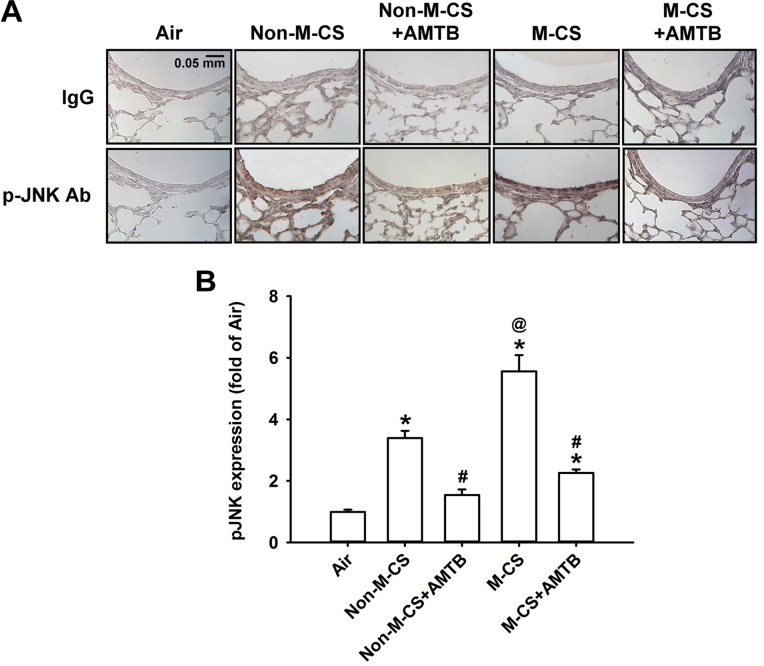
Menthol cigarette smoke induces higher lung epithelial expression of p-JNK than Non-M-CS does in mice with subchronic exposure. p-JNK represents phosphor-c-Jun N-terminal kinase. See Figure 2 caption for further explanations of the treatments in the five study groups of mice. **(A)** Representative images showing immunostaining with a p-JNK antibody of the lung sections obtained from the five groups of mice. Specificity of immunostaining was confirmed using an IgG-negative control. The magnification of each panel was 400×. **(B)** Quantification of the expression levels of epithelial p-JNK in the lung sections with immunostaining. Data in each group are mean ± SEM from six mice. ^∗^*p* < 0.05 vs. the air-exposed group; ^@^*p* < 0.05 vs. the Non-M-CS-exposed group; ^#^*p* < 0.05 vs. the same type of CS group with vehicle treatment. Note that the increased expression of epithelial p-JNK induced by M-CS was higher than that induced by Non-M-CS, but the increased expressions induced by both types of CS were significantly reduced to similar levels by treatment with AMTB.

**FIGURE 6:**
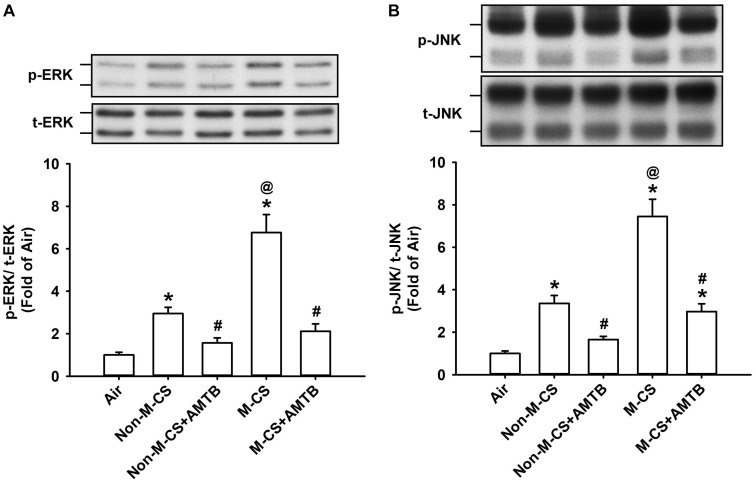
Treatment with a TRPM8 antagonist (AMTB) suppresses the increased lung expression of p-ERK or p-JNK induced by M-CS or Non-M-CS in mice with subchronic exposure. p-ERK **(A)** and p-JNK **(B)** represent phosphor-extracellular signal-regulated kinase and phosphor-c-Jun N-terminal kinase, respectively. t-ERK and t-JNK represent total proteins of these two kinases. See Figure 2 caption for further explanations of the treatments in the five study groups of mice. The protein levels in the lung tissues were analyzed by Western blot. Data in each group are mean ± SEM from six mice. ^∗^*p* < 0.05 vs. the air-exposed group; ^@^*p* < 0.05 vs. the Non-M-CS-exposed group; ^#^*p* < 0.05 vs. the same type of CS group with vehicle treatment. Note that the increased expression of lung p-ERK or p-JNK induced by M-CS was higher than those induced by Non-M-CS, but the increased expressions induced by both types of CS were significantly reduced to similar levels by treatment with AMTB.

As MAPKs, particularly ERK and JNK, are important for the induction of CS-induced lung inflammation (Liu et al., 2014; Lin et al., 2015, 2017), we subsequently studied the CS-induced increases in the expression of MAPKs in lung epithelium and lung tissues, as well as the suppressive effects of AMTB on these responses. Immunohistochemical analysis showed that the positive staining for p-ERK (Figure 4A) or p-JNK (Figure 5A) was stronger in the epithelial cells of the lung sections from the Non-M-CS-exposed or M-CS-exposed mice than in those from the air-exposed mice. Moreover, the positive staining for p-ERK (Figure 4A) or p-JNK (Figure 5A) was stronger in the epithelial cells of the lung sections from the M-CS-exposed mice than in those from the Non-M-CS-exposed mice. Quantitative analysis revealed that the increase in the relative expression level of p-ERK (Figure 4B) or p-JNK (Figure 5B) measured as positive epithelial cells by immunostaining was higher in the M-CS-exposed group than in the Non-M-CS-exposed group. Furthermore, Western blot analysis revealed that the expression of p-ERK (Figure 6A) or p-JNK (Figure 6B) was significantly higher in the lung tissues of the M-CS-exposed mice than in those of the Non-M-CS-exposed mice. These increased levels of expression of p-ERK (Figures 4, 6A) and p-JNK (Figures 5, 6B) induced by both types of CS were significantly reduced to similar levels after treatment with AMTB.

### Non-M-CS and M-CS Induce Similar Levels of Biomarkers of CS Exposure in Mice With Acute Exposure

**FIGURE 7:**
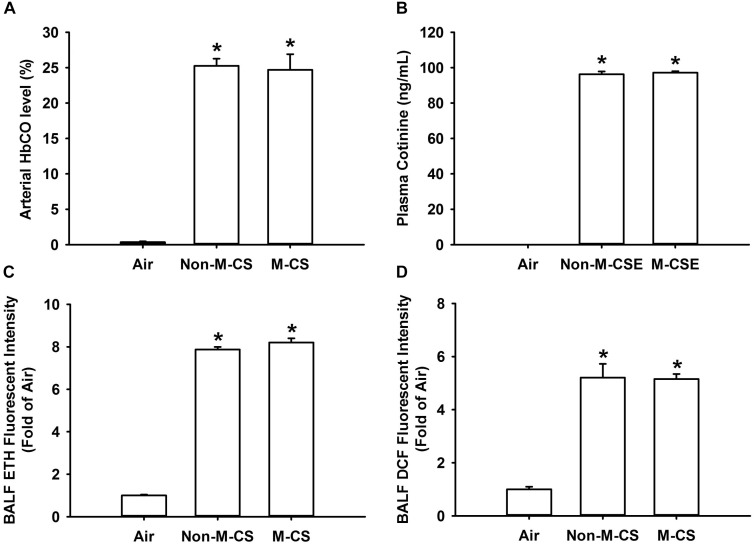
Menthol cigarette smoke and Non-M-CS induce similar levels of biomarkers of CS exposure in mice with acute exposure. Mice were acutely exposed to air, Non-M-CS, or M-CS for 20 min. Bronchoalveolar lavage fluid and blood samples were collected and analyzed immediately after air or CS exposure. **(A)** Arterial carboxyhemoglobin (HbCO) concentrations were analyzed by blood gas system. **(B)** Nicotine concentrations in the blood were assessed with an ELISA kit to detect the levels of cotinine, the predominant metabolite of nicotine. The levels of reactive oxygen species, particularly hydrogen peroxide and superoxide, were measured by DCFH-DA **(C)** or HE/ETH **(D)** fluorescent probe assay, respectively. ^∗^*p* < 0.05 vs. the air-exposure group. Data in each group are mean ± SEM from six mice. Note that no differences in levels of these biomarkers were detected between the M-CS and Non-M-CS groups.

To reflect the amounts of CS inhaled by mice during CS exposure, we measured several biomarkers of CS exposure after a 20 min acute exposure. We found that the M-CS exposure induced similar increases in arterial blood levels of HbCO (Figure 7A) and cotinine (Figure 7B) and BALF levels of ROS, particularly superoxide (Figure 7C) and hydrogen peroxide (Figure 7D). Additionally, the levels of these biomarkers measured in mice with either type of CS exposure were all significantly higher than those measured in air-exposed mice (Figure 7).

## Discussion

In this study, we clearly demonstrated that TRPM8 was expressed in the airway epithelium and lung tissues and that its expression was up-regulated by the subchronic exposure to Non-M-CS for 7 days (Figure 1). Additionally, the subchronic exposure to Non-M-CS induced lung inflammation, as evidenced by the pathohistological findings (Figure 2), increased lung vascular permeability (Figure 3A), pulmonary infiltration of inflammatory cells (Figures 3B,C), increases in BALF and lung levels of MIP-2 (Figures 3D,E), and increases in lung levels of 4-HNE (Figure 3F). The pulmonary infiltration of inflammatory cells is mainly due to increases in neutrophils and macrophages, both of which are important for the induction of Non-M-CS-induced lung inflammation (Barnes, 2004). MIP-2 and 4-HNE are important inflammatory chemokine (Thatcher et al., 2005; Liu et al., 2014; Lin et al., 2015) and biomarker of oxidative stress (Rahman et al., 2002; Liu et al., 2014; Lin et al., 2015), respectively, in Non-M-CS-induced lung inflammation. This Non-M-CS-induced lung inflammation in the current work was associated with increases in epithelial (Figures 4, 5) and lung expression (Figure 6) of p-ERK and p-JNK. This animal model was then used to compare the inflammatory effects of Non-M-CS and M-CS on the lungs and assess the role of TRPM8. We found that relative to Non-M-CS exposure, M-CS exposure induced greater increases in the above-mentioned lung inflammatory indices, as well as in the epithelial and lung expression of TRPM8, p-ERK, and p-JNK (Figures 1–6). More important, these increased inflammatory indices and expressions of epithelial and lung p-ERK and p-JNK induced by both types of CS were greatly suppressed to similar levels after treatment with a TRPM8 antagonist, AMTB (Figures 2–6). Collectively, these results suggest that M-CS induces more severe lung inflammation and more vigorous activation of MAPKs than Non-M-MCS does in mice with subchronic exposure. Moreover, TRPM8 plays an important role in these augmented effects of M-CS.

Several *in vitro* studies have shown that non-neuronal TRPM8 is expressed in lung epithelial cells and has inflammatory functions when activated by cold air or its ligand menthol (Sabnis et al., 2008a,b; Li et al., 2011; Lin et al., 2017). Lung epithelial cells are a direct target of CS, and these cells play a vital role in the initiation and progression of CS-induced lung inflammation (Mossman et al., 2006; Chung and Adcock, 2008). Therefore, we focused on the expression and function of lung epithelial TRPM8 *in vivo* in this study. We infused the TRPM8 antibody into the mouse airways for *en face* immunostaining, which resulted in a clear visualization of the airway geometry. TRPM8 was found to be expressed in the lung epithelium, as supported by the evidence from the immunohistochemical analysis of the lung sections subjected to *en face* immunostaining. We also showed that subchronic exposure to M-CS induced greater increases in the epithelial and lung expression of TRPM8 than the exposure to Non-M-CS did. These observations imply that the inflammatory functions of lung epithelial TRPM8, if any, may be amplified in response to CS exposure via this increased expression and that M-CS has a considerably potent effect on this amplification.

The mechanism in which M-CS induces more vigorous activation of MAPKs and severe lung inflammation than Non-M-CS does remains obscure. One possibility is that a part of MAPK activation and the inflammatory responses augmented by M-CS might have been caused by the additional stimulation of epithelial and lung TRPM8 by menthol in M-CS because this part was totally eliminated by treatment with AMTB. As ERK and JNK are known to be crucial signaling molecules for the induction of CS-induced lung inflammation (Mossman et al., 2006; Liu et al., 2014; Lin et al., 2015, 2017), the augmented lung inflammation induced by M-CS in this study might have resulted from a rigorous activation of the MAPKs. These *in vivo* findings are consistent with the observations from our recent *in vitro* study (Lin et al., 2017). In that study, we showed that M-CS extracts, compared with Non-M-CS extracts, produced more remarkable activation of MAPK signaling and interleukin-8 (IL-8) induction in human lung epithelial cells. Treatment with AMTB or complete knockout with TRPM8 prevented the part of the responses of MAPKs and IL-8 that were augmented by M-CS extracts (Lin et al., 2017). However, it should be noted that in this study, treatment with AMTB also reduced the MAPK activation and inflammatory responses induced by Non-M-CS and that no differences were observed in the residual responses to Non-M-CS and M-CS after blockade of TRPM8. This result suggests that a common stimulant generated by both CS types may also stimulate TRPM8, activate MAPK signaling, and induce lung inflammation. In this regard, ROS appears to be a plausible candidate of this stimulant because CS contains high concentrations of free radicals, radical precursors, and long-lived metastable radical intermediates, all of which may generate ROS (Pryor, 1992). This notion is supported by our recent *in vitro* study (Lin et al., 2017), which showed that ROS derived from Non-M-CS and M-CS extracts may also stimulate TRPM8 and lead to the activation of MAPKs and promotion of IL-8 induction in lung epithelial cells because these responses can be prevented by treatment with a ROS scavenger or AMTB. Other investigators have demonstrated that TRPM8 is sensitive to ROS in the uroepithelium with direct exposure to H_2_O_2_ (Nocchi et al., 2014). The same ROS mechanism involved in the stimulation of TRPM8 may possibly be at work in mice subchronically exposed to both types of CS. TRPM8 is also expressed in lung cells other than epithelial cells (Yang et al., 2006; Johnson et al., 2009; Journigan and Zaveri, 2013; Zhang et al., 2016), all of which may participate in the development of CS-induced lung inflammation (Chung and Adcock, 2008). Accordingly, the blockade of TRPM8 in these cells by AMTB could have contributed to the reduced lung inflammation that was observed in our *in vivo* model. Collectively, our results suggest that the involvement of TRPM8 is greater in the responses to M-CS than in the responses to Non-M-CS possibly because of the additional action of menthol in M-CS on TRPM8.

The other possibility to explain the more severe lung inflammation observed in this study is that M-CS-exposed mice could have inhaled a larger total volume of CS into their lungs relative to the Non-M-CS-exposed mice. This notion is based upon the findings that menthol, when given alone, can act on neuronal TRPM8 localized at the airway sensory nerve terminals (Fisher, 2011) and counteract airway irritation in animals (Willis et al., 2011; Liu et al., 2014) and humans (Millqvist et al., 2013). A previous investigation (Ha et al., 2015) reported that the acute exposure (for 20 min) of mice to CS with added menthol vapor resulted in a 1.5-fold increase in plasma levels of cotinine (a nicotine metabolite) over those observed in mice exposed to CS without added menthol vapor. This observation seems to imply that the mice inhaled a greater total amount of CS when menthol vapor was added to the smoke. However, whether the characteristics and concentrations of added menthol vapor are similar to those of menthol in M-CS remains debatable. In this study, we measured several biomarkers related to CS exposure and found that the blood levels of HbCO and cotinine, as well as the BALF levels of superoxide and hydrogen peroxide, in the mice acutely exposed (for 20 min) to M-CS did not differ from those in the mice exposed to Non-M-CS. In the case of acute exposure, blood levels of HbCO depend on the total amount of CO in inhaled CS (Jones et al., 1983), and blood levels of cotinine depend on the total amount of nicotine intake (Benowitz et al., 2009). Also, BALF levels of superoxide and hydrogen peroxide, two major types of ROS, may be directly related to the total amount of CS inhaled; this notion indicates that the insults to the airway epithelium by ROS in BALF are similar in mice exposed to these two CS types (Lin et al., 2015). Thus, we found no evidence of inhalation of a larger total volume of CS in mice with acute M-CS exposure relative those with acute Non-M-CS exposure. In clinical settings, the smoothing properties of menthol also promote the concept that in smokers, menthol cigarette smoking, relative to non-menthol cigarette smoking, may result in larger puffs, deeper inhalation, or longer retention time in the lungs, thereby producing more adverse effects (Hoffman, 2011; Lawrence et al., 2011; Besaratinia and Tommasi, 2015). However, results from studies on the smoking topography and blood biomarkers of CS exposure in smokers (Caraballo et al., 2001; Heck, 2009; Hoffman, 2011; Lawrence et al., 2011; Strasser et al., 2013) do not support this hypothesis. Hence, this possibility appears to be unlikely.

Non-M-CS has been shown to induce airway neurogenic inflammation mainly via activation of certain type of lung vagal afferents (Kou et al., 2011). Previous studies have demonstrated that TRPM8 is expressed in a subpopulation of vagal afferent neurons innervating lung tissues in rats (Xing et al., 2008), but not in mice (Nassenstein et al., 2008). The possible involvement of airway neurogenic inflammation in the M-CS-induced augmented lung inflammation observed in this study remains to be investigated.

This study has some limitations. First, our findings were derived from mice with subchronic CS exposure, and this model cannot represent the clinical scenario in smokers with chronic obstructive pulmonary disease. However, our findings may at least be extrapolated to the scenario of the early stage after initiation of smoking. Second, we only measured the levels of biomarkers after acute CS exposure. This is mainly due to the fact that, in the setting of subchronic CS exposure, menthol in menthol cigarettes can substantially decrease the absorption and/or increase the clearance of nicotine, thereby affecting the blood concentration of cotinine (Abobo et al., 2012). In addition, superoxide and hydrogen peroxide in BALF are short-lived ROS (Pryor, 1992) that are more precisely measured immediately after acute CS exposure, as compared to the situation in subchronic CS exposure. Third, non-menthol and menthol cigarettes from the same brand with similar characteristics (tar, nicotine, and tobacco contents; length; and diameter) are not commercially available. Therefore, we decided to use these two types of cigarettes with notably similar characteristics from two different brands.

In summary, we demonstrated that relative to the subchronic exposure to Non-M-CS, the subchronic exposure to M-CS induced greater TRPM8-mediated activation of MAPKs and lung inflammation in mice. The augmented inflammatory effects of M-CSE may be due to an additional stimulation of epithelial and lung TRPM8 by menthol in M-CS. Although the health effects of cigarette smoking are well documented, those of menthol cigarettes, in comparison with non-menthol cigarettes, are not widely studied (Hoffman, 2011). The findings of this study may provide support to the clinical observation regarding the association of menthol cigarette smoking with severe lung inflammation (Park et al., 2015).

## Ethics Statement

This study was carried out in accordance with the recommendations of the guidelines, Institutional Animal Care and Use Committee. The protocol was approved by the Animal Care and Use Committee of the National Yang-Ming University.

## Author Contributions

A-HL, M-HL, H-KK, and D-WP conducted the studies and analyzed and interpreted the data. A-HL and M-HL wrote the manuscript. T-SL and YK led the project, interpreted the data, and finished the manuscript.

## Conflict of Interest Statement

The authors declare that the research was conducted in the absence of any commercial or financial relationships that could be construed as a potential conflict of interest.
